# In Vitro Maturation of Oocytes Retrieved from Ovarian Tissue: Outcomes from Current Approaches and Future Perspectives

**DOI:** 10.3390/jcm10204680

**Published:** 2021-10-13

**Authors:** Chloë De Roo, Kelly Tilleman

**Affiliations:** Department for Reproductive Medicine, Ghent University Hospital, 9000 Ghent, Belgium; Kelly.Tilleman@UZGent.be

**Keywords:** in vitro maturation, OTO-IVM, CAPA-IVM

## Abstract

In vitro maturation (IVM) of transvaginally aspirated immature oocytes is an effective and safe assisted reproductive treatment for predicted or high responder patients. Currently, immature oocytes are also being collected from the contralateral ovary during laparoscopy/laparotomy and even ex vivo from the excised ovary or the spent media during ovarian tissue preparation prior to ovarian cortex cryopreservation. The first live births from in vitro-matured ovarian tissue oocytes (OTO-IVM) were reported after monophasic OTO-IVM, showing the ability to achieve mature OTO-IVM oocytes. However, fertilisations rates and further embryological developmental capacity appeared impaired. The introduction of a biphasic IVM, also called capacitation (CAPA)-IVM, has been a significant improvement of the oocytes maturation protocol. However, evidence on OTO-IVM is still scarce and validation of the first results is of utmost importance to confirm reproducibility, including the follow-up of OTO-IVM children. Differences between IVM and OTO-IVM should be well understood to provide realistic expectations to patients.

## 1. Introduction

The potential application of in vitro maturation (IVM) of oocytes as an alternative to in vitro fertilization (IVF) of in vivo-matured oocytes has gained increasing interest in the last two decades. IVM involves the in vitro maturation of immature cumulus–oocyte complexes (COCs) collected from antral follicles, with or without follicle-stimulating hormone (FSH) priming, from the germinal vesicle (GV) stage to metaphase II (MII) [1,2,3,4]. Since the first observation in 1934 by Pincus and Enzmann [5] of rabbit oocytes capable of undergoing spontaneous in vitro maturation and fertilization, IVM has successfully been carried out in different species, with Cha et al. [6] being the first to report, in 1990, an IVM birth in humans from immature oocytes retrieved from donors [6]. The first IVM birth from the mother’s own immature oocytes followed four years later [7]. The intriguing history of IVM has been summarized in 2018 by Shirasawa et al. [8] and more recently (2020) by De Vos M et al. [9].

Although promising, the rollout of IVM was initially hampered by the reduced oocyte maturation rate of this method. Subsequent improvements were made to optimize both in vitro cytoplasmic and meiotic maturation and the developmental competence of oocytes by means of preparatory treatments in patients and optimisation of laboratory protocols. One of the procedures explored to optimize maturation rate was FSH priming before oocyte retrieval [3,9]. For this purpose, patients were prepared with a short regime of FSH and triggered with human chorionic gonadotropin (hCG) and/or gonadotropin-releasing hormone (GnRH) agonist (termed hCG-primed IVM or truncated IVF in the first case, and truncated IVF without FSH in the second case) [3,9]. Likewise, laboratory protocols were optimized from monophasic or single-step IVM to biphasic IVM or pre-IVM to support the synchrony in cytoplasmic and nuclear maturation [10]. The biphasic protocol includes a pre-maturation culture or ‘capacitation’ (CAPA) which increases oocyte maturation potential [11]. This protocol is now known as ‘CAPA-IVM’.

IVM was initially introduced in clinical practice as a safer alternative for conventional ovarian stimulation to avoid ovarian hyperstimulation syndrome (OHSS) and ovarian torsion [12,13]. Thus, IVM constitutes a promising alternative to in vitro fertilization of in vivo-matured oocytes conventional in predicted or expected high responders, such as polycystic ovary syndrome (PCOS) patients [9,13,14,15,16]. Encouraging results have been reported in these patients, with maturation rates up to 84% [17], fertilization rates up to 80% [18], clinical pregnancy rates up to 50% per cycle [19] and live birth rates up to 33% [19] as reviewed by the Cochrane library in 2018 [13]. Subsequently, IVM indications have been broadened to include fertility preservation and rare conditions where a controlled ovarian stimulation failed to result in mature oocytes, such as resistant ovary syndrome or repeated deficient oocyte maturation [16,20].

Recently, other alternatives have been introduced in IVM related to the way follicles are retrieved. Although in its initial stages COCs were collected by transvaginal follicle aspiration, new procedures include in situ follicle aspiration from the ovary or the contralateral ovary during laparoscopy/laparotomy and ex vivo aspiration either from the excised ovarian tissue (OT) or the spent media during cortex preparation for cortex cryopreservation. This latter protocol, called ovarian tissue oocyte (OTO)-IVM [21], is now increasingly being explored to maximize fertility preservation potential where the clinical application of OT transplantation may be contraindicated. Cancer patients in whom orthotopic transplantation of OT carries the risk of malignant cell contamination [22] and transgender men in whom transplantation is undesired as it restores the endogen productions of female hormones [23] are targets potentially benefiting of OTO-IVM. We have conducted a review of the outcomes achieved with OTO-IVM until today and on the current evidence on CAPA-IVM. The role of this latter protocol in women undergoing OT follicle retrieval is further discussed.

## 2. Materials and Methods

A literature search of PubMed was performed using search terms containing to the method of interest (e.g., in vitro maturation or IVM alone or in combination with OTO, PCOS, ex vivo, CAPA, biphasic, ovarian tissue oocyte) and reproductive outcomes (e.g., live birth, pregnancy, outcome). No date restrictions were used. Papers were selected by the authors based on completeness of the reproductive outcome and fitting within the scope of this narrative review.

## 3. Results

### 3.1. Monophasic OTO-IVM

**Figure 1:**
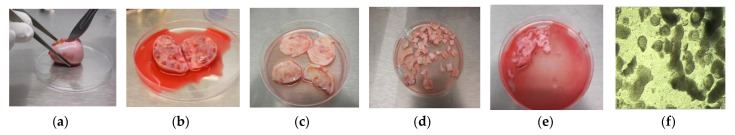
Ovarian tissue preparation for cryopreservation: (**a**) Bisection of the ovary; (**b**) Bisected ovary with antral follicles displayed in the medulla; (**c**) Cortical tissue of which the medulla is removed; (**d**) Cortical tissue pieces ready for cryopreservation; (**e**) Residual medulla after it has been mechanically scraped from the cortical tissue; (**f**) Microscopic view of the Petri dish (**e**) showing cumulus–oocyte complexes.

Given that oocytes can be aspirated by ultrasound from small follicles visible on the ovary, it was reasonable to assume that upon oophorectomy these small follicles and residing oocytes would be present in the ex vivo-collected OT. This protocol, known as OTO-IVM, was first described by Revel et al. [24], who reported the collection of immature oocytes from small antral follicles during the processing of OT. Since then, other authors have used this protocol to find an additional pool of oocytes that could maximize the preservation of fertility in patients undergoing oophorectomy in the case of urgent gonadotoxic therapies. The standard procedure for cryopreservation of OT comprises removing the medulla from the ovarian cortex and cutting the cortical tissue into smaller pieces before exposing them to freezing medium followed by a slow freezing or vitrification protocol (please consult Leonel et al. 2019 for review on cryopreservation of ovarian tissue [25]) (Figure 1). The residual medulla is usually discarded, although this disposed solution contains a considerable pool of immature oocytes that can be visualized and identified as COCs when the dish is carefully inspected under a stereo microscope.

In 2011, Kristensen et al. [26] described the possibility of collecting COCs from leftover medulla. Despite this, many centers still discard the medulla during OT cryopreservation [26]. Several studies have shown OTO-IVM maturation rates of 30–40% [21,23,27,28,29,30,31,32], although maturation rates up to 68% have been reported [29,33]. While the monophasic OTO-IVM protocol is alike in these studies, three of the studies showed that higher transportation times from procurement of the tissue to the IVF laboratory, in which the tissue is exposed to low temperatures, were related to lower, although not statistically significant, maturation rates [34,35,36].

Detailed literature on embryo development from OTO-IVM oocytes, especially up to the blastocyst stage, remains scarce (Table 1). Several case reports and small cohort studies have shown that the average rate of normal fertilised OTO-IVM oocytes–as characterised by the appearance of two pronuclei (2PN)—is lower than in vivo-matured oocytes obtained by conventional ovarian stimulation, ranging from 35.2% to 65% [24,27,28,29,35,37,38,39]. This wide range could be due to differences in the patient cohorts included in these studies. As commented before, patients with oncological diagnoses have been the main focus of studies on OTO-IVM, although transgender men are a new focus. It is worth noting that transgender men included in these studies are still under testosterone treatment at the time of oophorectomy. Additionally, in these studies, either fresh- or vitrification-warmed OTO-IVM oocytes were used, which is likely to have an impact on the fertilisation rates. Embryo development data are mostly related to Day 2 or Day 3, and only two studies show results up to the blastocyst stage. In a case report, Kirillova et al. [38] described the development of three blastocysts after intracytoplasmic sperm injection (ICSI) from 33 COCs retrieved from a woman with breast cancer using fresh OTO-IVM. Lierman et al. [39], describes the development of one blastocyt after ICSI out of 1903 COCs retrieved from 83 transgender men in vitrification-warmed OTO-IVM oocytes. The development of almost all embryos was arrested in this latter study, where COCs were collected at the time of gender-affirming surgery and while patients were under testosterone treatment. 

The results of these studies showed that, although the OTO-IVM oocytes were able to mature and to display a good morphological structure, their fertilisation and further embryological developmental capacities were somehow impaired. In any case, this low effectiveness has not withheld clinics to perform embryo transfers with OTO-IVM embryos and five live births have been reported so far [42,43,44] (Table 2). 

In summary, the OTO-IVM protocol seems promising and has resulted in healthy babies [42,43,44]. The OTO-IVM oocytes display a good morphological structure but fertilisation and further embryological developmental capacities seemed to be impaired, as shown by the low efficacy achieved in comparison to other IVF procedures. This is probably related to the COCs IVM process.

### 3.2. Biphasic IVM

Oocyte maturation is a complex event achieving both nuclear and cytoplasmic maturation. The pathways involved in oocytes maturation have been reviewed in detail by Coticchio et al. [45]. In summary, nuclear maturation consists of chromosomal condensation, segregation and polar body extrusion, whereas cytoplasmic maturation involves redistribution of organelles and dynamic changes in cytoskeletal filaments [46]. The lower developmental capacity of in vitro-matured oocytes is related to an asynchronic cytoplasmic and nuclear maturation. In vivo, a meiotic arrest provides the necessary time for cytoplasmic maturation, which is resumed after the lutheinizing hormone (LH) surge. LH receptor activation causes the up-regulation of the epidermal growth factor (EGF)-like family [47]. Amphiregulin (AREG), the most abundant EGF-ligand is present in follicular fluids of mature follicles. AREG cannot be detected before the LH surge or before human chorionic gonodotrophin (hCG) trigger and depletion of AREG was accompanied with immaturity of the oocytes concluding that AREG is a useful marker and important modulator of oocyte maturity and competence [48]. During in vitro maturation, granulosa cell–COC communication is important. Granulosa cells secrete certain factors, amongst them C-type natriuretic peptides (CNPs). CNP plays a role as maturation inhibitor maintaining the meiotic arrest. In response to the LH surge, meiosis resumes by decreasing the expression of CNP. Decreased levels of CNP can be found in murine ovaries and human follicular fluid [49]. When immature oocytes are isolated and cultured in vitro, oocytes spontaneously resume meiosis because the inhibitory environment is lost.

The culturing of COCs in media containing these secreted CNPs has been shown to improve the efficacy of the IVM in several animal species, thus supporting the role of CNP in meiosis arrest, which is conserved among mammals. Indeed, in vitro maturation rates of COCs obtained from ex vivo goat [50] or bovine ovaries [51] were significantly increased when an extra step containing CNP was performed in combination with standard IVM. The developmental capacity of these two-step IVM oocytes was dramatically increased, resulting in higher blastocyst rates and a higher blastocyst cell content in both animal studies [50,51]. This superior developmental capacity is the result of a temporarily delay in spontaneous nuclear maturation which prolongs the time of the cytoplasmic maturation by improving the mitochondrial function [51,52]. A recent study has provided evidence on the effect of treatment of immature cattle oocytes with CNP before IVM on the patterns of mitochondrial location, increasing the mitochondrial content and decreasing reactive oxygen species (ROS) during IVM [52]. Clearly, CNP impacts the level of cAMP, which is important for maintaining the oocytes in meiotic arrest.

EGFs are important for periovulatory events, including the re-initiation of meiosis in oocytes. In vivo, the LH surge induces the release of EGF from the granulosa cells. Studies conducted in the early 1990s showed that 30 h of EGF supplementation increased the amount of MII oocytes from cumulus stripped GV oocytes from 33.9% to 64.3%. When COCs stayed intact, similar amounts of metaphase II (MII) oocytes were achieved; however, a higher fertilisation rate was noted (71.7% vs. 45.6%, *p* < 0.05) [53]. Thus, these studies provided evidence of the benefit of EGF supplementation on the nuclear and cytoplasmic in vitro maturation of COCs [53]. Amphiregulin or AREG, a member of the EGF member family is present in pre-ovulatory follicles and the optimal amount of AREG, determined in rhesus monkeys, showed that 10 ng/mL was more beneficial than 100 ng/mL for oocyte maturation [54]. AREG promotes the maturation of human GVs in vitro (from 36.5% to 75.5%), resulting in an increased number of normal fertilised oocytes after ICSI [55]. AREG, in the presence of gonadotrophins, appears to accelerate and facilitate germinal vesicle breakdown and MI–MII transition [54]. This can be found abundantly in follicular fluid from patients undergoing IVF [56]. Biphasic IVM of ex vivo-collected ovine COCs combing CNP in a first step followed by AREG supplementation in a second IVM step has shown to dramatically increase the blastocyst rate [57].

### 3.3. Biphasic IVM in Human

The proof of concept for the biphasic IVM protocol in animal studies, which showed its potential for maturing COCs collected ex vivo from unstimulated ovaries [50,51,52,54], encouraged its use in humans [10]. Table 3 summarises the studies conducted using CAPA-IVM vs. monophasic IVM in humans. Detailed information of the laboratory protocol and the media used are provided in these papers. In summary, monophasic IVM was carried out in IVM medium (IVM system, Medicult, Origio), supplemented with 75 mIU/mL HP-hMG (Menopur), 100 mIU/mL hCG (Pregnyl) and 10 mg/mL human serum albumin (HSA). Cumulus complexes were cultured for 30 h in groups of 10 COCs per well in 500 µL IVM medium with oil overlay at 37 °C, 6% CO_2_ in air. Biphasic IVM was performed in two steps: the first step contained IVM medium (IVM system, Medicult, Origio), supplemented with 1 mIU/mL rFSH (Puregon), 5 ng/mL insulin, 10 nM estradiol, 10 mg/mL HSA and 25 nM CNP (Tocris Bioscience; Abdingdon, UK). COCs were cultured in groups of 10 in 500 µL of this medium under oil for 24 h at 37 °C, 6% CO_2_ in air. Following this 24 h incubation, COCs were washed and transferred to the 2nd step, containing Medicult IVM medium supplemented with 5 ng/mL insulin, 10 nM estradiol, 100 ng/mL human recombinant Amphiregulin (rhAREG, Tocris Biosciences) and 100 mIU/mL rFSH, and incubated for 30 h under the same conditions.

This biphasic technique was first used in the patient cohort most intensively studied in, and benefiting from, IVM: PCOS patients. Biphasic IVM was initially clinically performed following the so-called ‘mild stimulation IVF’, in which a short treatment of gonadotropins is performed prior to ultrasound-guided oocyte aspiration [10]. This approach has been shown to improve oocyte maturation (up to 70%), resulting in higher fertilisation rates (up to the 2PN rate of in vivo-matured oocytes) and yielding higher amounts of embryos and blastocysts (up to 23% blastocyst rate) compared to the monophasic protocol [11]. A pilot study conducted in Belgium translated the CAPA-IVM protocol derived from animal studies to human. The results of this study were subsequently confirmed in a prospective study conducted by Sánchez et al. [58] in Vietnam. The report of the first 20 babies born showed that the protocol was efficacious and safe in short-term follow-up [59]. As shown in Table 3, this protocol has allowed higher amounts of in vitro-matured oocytes, higher fertilisation rates after ICSI and higher blastocyst rates to be achieved compared to the monophasic protocol [11,38,58,59]. Sánchez et al. [58] have recently reported the birth of healthy children using CAPA-IVM. DNA methylation studies and analysis of imprinting genes showed no differences in embryos derived from CAPA-IVM and ovarian stimulation adding more evidence on the safety of the protocol [60].

The basis for clinical application of CAPA-IVM mostly stems from evidence of COCs collected through ultrasound-guided aspiration from ovary after mild ovarian stimulation. Although animal studies have provided evidence on the possibility of using this protocol in unstimulated ovaries, this path has only recently been explored in humans [38]. A first human pilot study using CAPA-IVM in COCs retrieved ex vivo from unstimulated ovaries showed an improvement in maturation and developmental capacity of the OTO-IVM oocytes compared to the monophasic protocol, thus providing a proof of concept for using CAPA-OTO-IVM in humans [38] (Table 3). This new path opens future possibilities for making the most of OTO-IVM in COCs retrieved in patients where oophorectomy has to be performed.

**Table 3:** Human studies comparing monophasic IVM vs. biphasic IVM.

Study Type	Methodology	Results	Reference
Prospective pilot study *n* = 15 PCOS patients age mean ± SD 28.9 ± 3.9 years Single center	Stimulation: 3 days HP-hMG (225 IU, 225 IU, 150 IU) (+2 days if applicable) COC retrieved via aspiration Sibling oocyte study: Monophasic IVM (*n* = 264 COCs) vs. biphasic IVM (*n* = 117 COCs)	COCs collected via ultrasound-guided in situ ovarian aspiration Monophasic vs. biphasic IVM: Maturation rate: 48% vs. 70% (*p* < 0.001) Fertilization rate: 62% vs. 82% (*p* < 0.001) Yield good quality embryos day 3: 23% vs. 43% (*p* < 0.001) Blastocyst rate: 14% vs. 23% (*p* < 0.05)	Sanchez F. et al., 2017 [11]
Prospective pilot study *n* = 40 PCOS patients Single center	Stimulation: 3 doses of 150 IU rFSH (no HCG trigger) COC retrieved via aspiration Sibling oocyte study: Monophasic IVM: (*n* = 20 patients; age: mean ± SD 28.8 ± 2.9 years; *n* = 238 COCs) vs biphasic IVM (*n* = 20 patients, age: mean ± SD 29.1 ± 3.4 y; *n* = 305 COCs)	COCs collected via ultrasound-guided in situ ovarian aspiration Monophasic vs. biphasic IVM: Maturation rate: 48% vs. 62% (*p* < 0.05) Fertilization rate was similar Yield good quality embryos day 3: 24% vs. 38% (*p* < 0.05) Blastocyst rate: 14% vs. 23% (*p* < 0.05) 1.8x higher amounts of usable embryos per patient: 2.2% vs. 4.2% (*p* < 0.001)	Sanchez F. et al., 2019 [58]
Prospective pilot study *n* = 80 PCOS patients Single center	Stimulation: 3 doses of gonadotropins (2.5days; ~377 IU used) (no HCG trigger) COC retrieved via aspiration Sibling oocyte study: Monophasic IVM: (*n* = 40 patients; age: mean ± SD 28.1 ± 3.1 years; *n* = 16.5 COCs/patient) vs biphasic IVM: (*n* = 40 patients; age: mean ± SD 28.5 ± 3.4 years; *n* = 17.5 COCs/patient)	COCs collected via ultrasound-guided in situ ovarian aspiration Monophasic vs. biphasic IVM: Maturation rate: 49% vs. 63.6% (*p* < 0.001) Fertilization rate similar Yield good quality embryos day 3: 26.8% vs. 30% Vitrified embryos after fresh ET: 1.3 ± 1.9 vs. 2.5 ± 2.5 (*p* < 0.05) Clinical pregnancy rate: 37.5% vs. 60% (NS; *p* = 0.06) 19 babies born after biphasic IVM no serious adverse events or reactions found Normal physical appearances were found in the newborn babies Detailed follow-up of the babies is being conducted.	Vuong L. et al., 2020 [59]
Prospective pilot study *n* = 10 patients (mean ± SE 29.40 ± 1.76 years; Range: 16–36 years) with gynecological malignancies Single center	Stimulation: 3 doses of 150 IU rFSH (no HCG trigger) COC retrieved via aspiration Sibling oocyte study: Monophasic OTO-IVM (*n* = 96 COCs) vs. biphasic OTO-IVM (*n* = 105 COCs)	COC identification via microscopic evaluation after tissue manipulation Monophasic OTO-IVM vs. biphasic OTO-IVM: Maturation rate: 35% vs. 56% (*p* < 0.05) Fertilization rate: 68.4% vs. 80% (*p* < 0.001) Yield good quality embryos day 3: 24% vs. 38%Blastocyst rate: 0% vs. 16% 1.8x higher amounts of usable embryos per patient: 2.2% vs. 4.2% (*p* < 0.001)	Kirillova A. et al., 2021 [38]

Polycystic ovary syndrome (PCOS); highly purified-human menopausal gonadotropin (HP-hMG); ovarian tissue oocyte in vitro maturation (OTO-IVM); recombinant follicle-stimulating hormone r(FSH); human chorionic gonadotropin (HCG); cumulus–oocyte complex (COC); standard deviation (SD).

## 4. Discussion

Several studies reporting ongoing pregnancies and live births after OTO-IVM have provided evidence on the potential of this methodology for the optimization of fertility potential. The first live births from OTO-IVM embryos were reported using the monophasic protocol, which clearly showed the ability of this protocol to achieve mature OTO-IVM oocytes. However, fertilisation rates and further embryological developmental capacities of the resulting oocytes seemed to be impaired.

The introduction of the biphasic IVM (or CAPA-IVM) protocol, where nuclear and cytoplasmatic maturation is enhanced, has been an important step forward in the COCs maturation protocol. In CAPA-IVM, the meiosis of immature COCs was arrested using CNP in the capacitation medium before moving COCs into a meiosis-promoting medium, which contains AREG in order to achieve both nuclear and cytoplasmic maturation. When trying to transpose the CAPA-IVM protocol to OTO, it should be noted that evidence supporting this methodology (and IVM in general) stems from highly respondent patients (PCOS patients) in whom COCs had been retrieved by in situ ovarian transvaginal aspiration. This is important, since overall maturation rates after OTO-IVM have shown to be lower than after IVM [24,27,28,29,35,37,38,39]. Besides this, other important aspects are to be taken into account to have realistic expectations when considering CAPA-IVM for OTO.

First, the differences in collection methods lead to COCs retrieved from a different follicle size. While COCs collected by transvaginal follicle aspiration are obtained from larger and, hence, more mature follicles, COCs retrieved ex vivo from an excised OT or spent media during cortex preparation come from very small, thus more immature, follicles. This results in a higher IVM rate in the first case, some adjustments to the IVM laboratory protocol will eventually be required.

Secondly, patient cohorts where OTO-IVM has been studied are particularly heterogeneous, comprising mainly oncological patients, but more recently also transgender men. OT cryopreservation has become standard of care for fertility preservation in pre-pubertal girls. This population is highly heterogeneous in terms of hormonal profile and ovarian constitution [27]. It has been hypothesized that pre-pubertal ovaries need a maturation phase to obtain optimal follicle function [27,61,62], which would imply the difficulty of harvesting COC in vitro or obtaining COC at all, these will show low maturity (18–33%) [27,32,63]. When performing COC retrieval after gender confirmation testosterone treatment, OTO-IVM showed that transgender men have lower developmental ability compared with cancer patients [39]. This may indicate that long-term testosterone treatment has an adverse effect on the development of oocytes [39]. For this reason, prior OT cryopreservation testosterone withdrawal can be recommended.

Thirdly, conversely to IVM, patient cohorts where OTO-IVM has been studied are heterogenous, these are comprised of mainly oncological patients, but more recently also transgender men. OTO-IVM has shown low developmental capacity in transgender men when compared to oncologic patients when COC retrieval is performed after gender-affirming testosterone treatment [39]. This might indicate a deleterious effect of prolonged testosterone treatment on the development competence of oocytes [39]. For this reason, testosterone withdrawal prior to the cryopreservation of OT is recommended. OT cryopreservation has become the standard of care for fertility preservation in prepubertal girls. This population is highly heterogeneous in terms of hormonal profile and ovarian constitution [27]. It has been hypothesized that prepubertal ovaries need a maturation phase to obtain optimal follicle function [27,61,62], which would imply the impossibility of harvesting COCs ex vivo or, if COCs could be obtained, whether these would present with lower maturation capacity (18–33%) [27,32,63].

**Figure 2:**
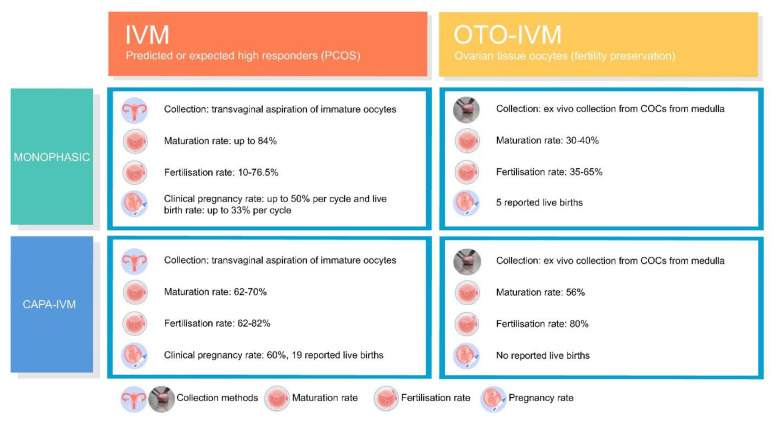
Overview of success ratios achieved with current IVM methods [11,13,27,28,35,38,42,43,44,58,59].

Figure 2 aims to provide objective information about the effectiveness of current IVM methods and chances, so that shared, informed decisions can be made.

Biphasic IVM has been clinically performed with so-called ‘mild stimulation IVF’ (i.e., with a short treatment of gonadotropins prior to ultrasound-guided oocyte aspiration), as this approach has shown to improve oocyte maturation rates, fertilisation rates and to result in higher amounts of embryos and blastocysts. A proof of concept for using biphasic OTO-IVM [38] provides new options for patients needing urgent fertility preservation. FSH priming is not always feasible or safe when the timeframe needed is impossible or when FSH priming appears to be unsafe given the underlying diagnosis. 

The analysis and follow-up of newborn children after OTO-IVM from immature oocytes from PCOS patients has provided evidence of the safety of this protocol [9,59,64]. It remains unknown if this evidence in enough to guarantee the safety of OTO-IVM. In this context, some concerns have been expressed regarding possible epigenetic risks for IVM children [9]. Despite promising initial reports, evidence on OTO-IVM is still scarce and validation of the first results is of utmost importance. A multicentric study confirming the reproducibility is necessary to validate the technique and outcomes in larger cohorts. Special attention should be given to the efficacy in the different patient populations using this approach. A correct follow-up of OTO-IVM children is mandatory, including the (epi)genetic safety of this laboratory protocol. 

## 5. Conclusions

OTO-IVM seems promising to optimize fertility potential, with several reports providing evidence of ongoing pregnancies and live births. IVM protocols have been studied in predicted or expected high responders. Success rates after OTO-IVM are not comparable to the latter, mostly due to differences in COC collection methods and in the patient populations used for these methodologies. These differences should be well understood in order to advise patients correctly and to stimulate research to introduce laboratory protocol adjustments. Although promising, evidence on OTO-IVM is still scarce and validation of the first results is of utmost importance to confirm reproducibility, including the follow-up of OTO-IVM children.

## Figures and Tables

**Table 1:** Embryological and reproductive outcomes of human monophasic OTO-IVM.

Study Type	Methodology	Results	Reference
Case report	43 years old patient Endometrial carcinoma Removal of polycystic ovary 10,000 IU HCG, 36 h prior to surgery, no FSH priming	Follicle aspiration from the ovary ex vivo (17 gauge needle) 17x oocytes retrieved, of which already 2x MII 3x MII after 24 h OTO-IVM; 1x 2PN after ICSI 7x MII after 48 h OTO-IVM; 3x 2PN after ICSI 40% 2PN rate; 4x day 3 embryos cryopreserved (2x ≥ 6 cell)	Revel A. et al., 2004 [24]
Case report	38 years old patient Ovarian adenocarcinoma	Follicle aspiration from the ovary in situ (26 gauge needle) during surgery 3x COCs identified 2x MII after 30 h OTO-IVM; vitrification After warming, 1x 2PN after ICSI 50% 2PN rate; 1x day 2 embryo (2 cell); no pregnancy after ET	Fadini R. et al., 2012 [40]
Cohort study	255 patients (mean ± SE 22.3 ± 1.26 years, range 10–40 years), different types of IVM 56 patients underwent OTO-IVM Hodgkin (*n* = 15), hematological cancer (*n* = 8), sarcoma (*n* = 11), breast cancer (*n* = 4), cervix cancer (*n* = 6), other (*n* = 12)	Follicle aspiration from the ovary ex vivo using needles and medium was analyzed microscopic after manipulation of the tissue 6.95 ± 0.83 COCs identified (mean ± SE) In 2.47 ± 0.43 MII after 48 h OTO-IVM; vitrification or ICSI on fresh oocytes 10/16 2PN after ICSI 62.5% 2PN rate; 1.67 ± 0.56 embryos cryopreserved	Hourvitz A. et al., 2015 [28]
Cohort study ^1^	9 patients (mean ± SD 30.6 ± 3.84 years, range 26–36 years) Breast cancer (*n* = 5), BRCA (*n* = 1), arteriovenous malformation (*n* = 1), systemic lupus erythematosus (*n* = 1), immature teratoma of tuba (*n* = 1)	COC identification via microscopic evaluation after tissue manipulation 10.8 ± 2.5 COCs identified (mean ± SEM) 4.6 ± 1.2 MII after 24 h or 40 h OTO-IVM 3.5 ± 1.0 2PN after ICSI 65 ± 11% 2PN rate 2.9 ± 0.6 embryos day 3 vitrified	Segers I. et al., 2015 [27]
Case series report	6 patients (mean 29 years, range 19–39 years) Endometrial (*n* = 2), ovarian (*n* = 3), double primary endometrial and ovarian cancer (*n* = 1)	Follicle aspiration from the ovary ex vivo (18 gauge needle) 53x COCs identified (mean 10.6 per patient) 36x MII after 48 h OTO-IVM; Vitrification of 28 MII oocytes for 4 patients, 5x embryos vitrified for 1 patient after ICSI on fresh OTO-IVM oocytes (8x MII injected); 62.5% 2PN rate	Park C.W. et al., 2016 [29]
Cross sectional study	136 patients (mean ± SD 27.6 ± 5.6 years) Different oncological indications (*n* = 120), hematological benign (*n* = 8), immunological disorders (*n* = 8)	Follicle aspiration from the ovary ex vivo, discarded tissue filtered through a cell strainer and microscopic evaluation 559x COCs subjected to OTO-IVM 145x MII after 24 h or 48 h OTO-IVM; Vitrification of 139x MII oocytes for 72 patients, 7x 2PN vitrified for 1 patient after ICSI on fresh OTO-IVM oocytes (12x MII injected in 5 patients); 58.3% 2PN rate 9x MII vitrification-warmed, 2x MII intact after warming, 1x embryo (low quality day 2); biochemical pregnancy after ET	Fasano G. et al., 2017 [35]
Case report	37 years old patient Mullerian adenocarcinoma Ovarian wedge resection (2 × 2 cm)	Follicle aspiration from the ovary ex vivo (20 gauge needle) 3x COCs identified 2x MII after 48 h OTO-IVM; 2x 2PN after ICSI; 100% 2PN rate 2x embryos (grade B and C day 3) vitrified	Mohsenzadeh M. et al., 2017 [37]
Cohort study ^1^	8 patients (mean ± SD 27.3 ± 4.8 years range 20–34 years)	Oocyte aspiration from the ovary ex vivo 25x COCs identified 12x MII after 36 h OTO-IVM 5x embryos cryopreserved after ICSI 5x embryos for ET in different patients: no pregnancies after ET	Kedem et al., 2018 [21]
Case report	30 years old patient Breast cancer Ovarian wedge resection (2 × 2 cm)	Follicle aspiration from the ovary ex vivo (21 gauge needle), remaining fluid after manipulation underwent microscopic evaluation 33x COCs identified 11x MII after 48 h OTO-IVM; 6x 2PN after ICSI; 54.5% 2PN rate 6x cleavage embryos (day 3), 3 blastocysts (euploid) vitrified	Kirillova A. et al., 2020 [38]
Prospectiveobservational study	12 patients (mean ± SD 27.3. ± 4.8 years range 20–34 years) Breast cancer (*n* = 6), B-cell lymphoma (*n* = 1), Hodgkin lymphoma (*n* = 2), cervical embryonal rhabdomyosarcoma (*n* = 1), pleiomorphic xanthoastrocytoma (*n* = 1), Ewing’s sarcoma (*n* = 1) Unilateral ovarian resection	Follicle aspiration from the ovary ex vivo (21 gauge needle), remaining fluid after manipulation underwent microscopic evaluation 37x COCs identified 14x MII after 24 h OTO-IVM; 8x MII were vitrified 4x 2PN vitrified for 3 patients after ICSI on fresh OTO-IVM oocytes (6x MII injected in 3 patients); 66.6% 2PN rate	Dietrich J. et al., 2020 [41]
Cross sectional study	83 patients (median; min-max: 20 years (17.6–38.4 years) Transgender patients Bilateral oophorectomy at the time of gender affirming surgery under testosterone treatment	COC identification via microscopic evaluation after tissue manipulation 1903x COCs identified 453x MII after 48 h OTO-IVM; 410x MII were vitrified, 208x MII warmed 48x 2PN (from 151 warmed intact) after ICSI with 1 sperm donor 34.5% 2PN rate 25x embryos day 3, 1x blastocyst day 5 (4BB) (euploid) 44x embryos arrested (91.7%)	Lierman S. et al., 2021 [39]

^1^ Only data from patients with embryologic developmental outcomes are summarized in the table; oocyte–ovarian tissue oocyte in vitro maturation (OTO-IVM); human chorionic gonadotropin (HCG); follicle-stimulating hormone (FSH); cumulus–oocyte complex (COC); intracytoplasmic sperm injection (ICSI); standard error (SE); standard error of the mean (SEM); standard deviation (SD); metaphase 2 oocyte (MII); normally fertilized zygote showing 2 pronuclei (2PN).

**Table 2:** Live births from monophasic OTO-IVM.

Study Type	Methodology	Results	Reference
Case report	21 years old patient Oophorectomy for bilateral large pelvic masses, peritoneal disease and ascites and raised serum CA125 of 1.279 U/mL left salpingo-ophorectomy at day 5 of the menstrual cycle	Follicle aspiration from the ex vivo ovary (18 gauge needle), No data on COCs collection, number of MII oocytes or embryo development. 24 h OTO-IVM performed 3x day 2 embryos cryopreserved by slow rate freezing. 2x embryos thawed, ET in artificial frozen-embryo transfer cycle Outcome: Healthy male singleton, 2.580 g	Prasasth. et al., 2014 [42]
Case report	23 years old patient 8 cm complex mass in the solitary left ovary Left salpingo-oophorectomy	Oocyte aspiration from a healthy piece of ovarian tissue (2 × 3 cm) 10x COCs identified 4x MII after 24 h OTO-IVM 4x 2PN after ICSI, 3x 2PN were cryopreserved by slow freezing 3x 2PN were thawed, DET (cleavage stage embryos) Outcome: Healthy male singleton, 3.883 g	Uzelac et al., 2015 [43]
Cohort study ^1^	12 patients (mean ± SD 29.8. ± 5.05 years range 23–36 years) Breast cancer (*n* = 9), Hodgkin lymphoma (*n* = 2), arterio-venous malformation of the uterus (*n* = 1)	COC identification via microscopic evaluation after tissue manipulation 157x COCs identified 51x MII after 28 h or 40 h OTO-IVM 24x MII vitrified 37x fresh OTO-IVM MII ICSI: 21x 2PN (56% 2PN rate) 15x embryos day 3 (good quality) vitrified Outcomes: 7x embryos warmed for 5 patients; 2 healthy female singletons (2.660 g and 3.860 g) for 2 patients 12x vitrification-warmed oocytes used for 2 patients; 1 healthy male singleton, 3.150 g	Segers et al., 2020 [44]

^1^ Only data from patients having embryo transfer are summarized in the table. The live birth reported by of Kedem et al. [21] has not been included since the paper described data from different types of IVM (not only OTO-IVM) and the live birth reported resulted from a PCOS patient where the IVM oocytes were aspirated from the ovary in situ. Cumulus–oocyte complex (COC); metaphase 2 oocyte (MII); oocyte–ovarian tissue oocyte in vitro maturation (OTO-IVM); embryo transfer (ET); normally fertilized zygote showing 2 pronuclei (2PN); dual embryo transfer (DET).
